# Causes Retarding Dental Progress

**Published:** 1871-05

**Authors:** G. E. Corbin


### CAUSES RETARDING DENTAL PROGRESS.

BY G. E. CORBIN, M. D.

I assume the position that I exist, and that I did not create myself; that thousands of other objects about me, both animate and inanimate, from the most minute to the most ponderous within the range of human vision, exist, and that they did not create themselves; hence, I infer the existence of an all wise, and all powerful Creator.

I assume the position that such a Creator will subject the objects of his creation, to the control of perfect and *immutable* laws.

So intimate is the relation existing between these objects—so great the dependence of one upon each and all the rest, the blade of grass upon the dew drop, our earth upon each of its fellow planets and the sun, that no single law of nature can be altered or for one moment suspended, without reducing the whole to the incomprehensible condition of a chaotic mass. Machinery like this, the possessions of an all wise Creator, will certainly ever be placed beyond the possibility of being tampered with, by erring, finite mortal man.

In other words, it is utterly impossible for man to violate, accelerate, retard, change or alter in the least degree a single law of nature.

It is just as much in conformity to nature's laws that a man who, from a state of beastly intoxication, passes a severe winter's night in a gutter, shall be frozen and loose his limbs or his life in consequence, as it is that a man who drinks cold water, retains his senses, and sleeps in a comfortable bed, shall escape such a calamity.

Man can not force water to run up hill to turn his mill; but, in its downward course, in obedience to the force of the attraction of gravitation, the ingenuity of man may trust in the buckets of his water-wheel, and thus utilize the power nature has supplied. He can do nothing more.

Nature is our only school—our only instructor; and he who possesses the most extensive and accurate knowledge of natural laws, is best fitted to discharge the duties and enjoy the pleasures of life.

As the longest life is far too short for the most profound intellect to grasp any considerable portion of the wisdom of nature, by mutual consent the field has been divided and subdivided, and allotted in sections to persons according to their choice of occupation.

A thorough knowledge of the minute structures, functions, etc., of plants in general, constitutes a botanist.

A knowledge of the action which the ultimate particles of matter exert upon each other, constitutes a chemist.

A knowledge (obtainable only by dissection) of all the apparent properties of organized bodies, constitutes an anatomist.

A physiologist is one who is familiar with the functions of animals and vegetables; that is, with the phenomena, the aggregate of which constitutes life.

A knowledge of so many of the general principles of botony, chemistry, and other kindred sciences as are applicable to his pursuit, constitutes an agriculturist.

A knowledge of chemistry, anatomy, physiology, pathology and numerous other kindred sciences, constitutes a physician.

In addition to the general knowledge necessary for the successful practice of medicine, a dentist should possess no inconsiderable mechanical experience in the use of instruments, and the working of metals under varying circumstances.

Before assuming that *any* causes are at present retarding the progress of dental science, it becomes necessary to briefly inquire into the history of its origin and subsequent development.

"The practice of medicine must have everywhere arisen from the accidents and infirmities to which mankind are liable. Some rude appliances to wounds and injuries, some equally rude observances in cases of internal disease, are common among the most barbarous people. The idea that disease is caused by the anger of superior and invisible beings, placed its treatment in the hands of the priests, and the same idea caused that treatment to consist mainly of superstitious rites."

Previous to the time when intercourse with Greece became common, the attempts made at Rome to cure diseases, for a period of six hundred years, according to Pliny, consisted mainly in superstitious observances; such as charms, incantations, the erecting of a temple to Apollo to stay the ravages of a pestilence, celebrating public games, driving a nail into the capitol, etc.

Though this is the record of the proceedings of a people who inhabited this earth more than two thousand years ago, similar practices *now* prevail among our North American Indians and other barbarous people.

During the primitive ages of the world, anatomy was not cultivated as a science, and hence, the art of surgery was necessarily undeveloped.

In later ages, when science was appreciated, and medical men felt the want of a correct knowledge of the human organism and its physiological characteristics, religious scruples forbade the opening of the human body to inspect the viscera.

Galen, even, with all his notoriety for medical attainments, so late in the history of our world, as the second century of the Christian era, was confined to the dissection of the inferior animals for the prosecution of his anatomical studies.

Following Galen's time, for nearly one thousand years, during the so-called middle ages, the practice of the healing art being invariably in the hands of priests, whom a canon of the church forbade to shed blood, surgical operations commonly fell into the hands of an inferior and ignorant class of barber surgeons, who frequently were itinerants.

Mandini di Luzzi, Professor of Anatomy at the University of Bologna, first publicly dissected two human bodies in the presence of medical students, in 1315; but not until the sixteenth century, did the study of human anatomy from actual dissection become general in the medical schools, and then in the schools of Italy.

As superstition gradually gave way before the dawning of science, that is, the unfolding of natural laws, remedial agents began to be administered according to a knowledge of cause and effect; and then it was that the foundations of the professions of medicine, surgery, and dentistry were laid.

It is less than a century since the art of dentistry has taken the rank of a distinct profession.

From advertisements in the newspapers of 1703, the practice of the art of making teeth and cleaning them, appears to have been in the hands of silversmiths or jewelers.

Artificial teeth at this period, like those of the Egyptians in the time of Herodotis, twenty-three centuries ago, were probably carved from wood or ivory; as we do not find it upon record that the manufacture of porcelain teeth was even suggested prior to the year 1728. Teeth of this material were first actually produced, though necessarily crude in shape and color, in France, in the year 1776.

To American industry and culture, *during the last quarter of a century*, is to be attributed the very near approach to perfection, now manifested in the manufacture of porcelain teeth.

As the progress of dental science during the past century, and especially during the last quarter of a century, has been so much more rapid than at any previous time during its history, the wisdom displayed in the selection of my subject may be thought very questionable.

It appears in evidence, however, that the development of the different branches of the healing art, as is likewise true of all other sciences, has held a direct relation to the intelligence of the period.

I maintain, therefore, that the causes, or rather *the* cause now retarding the progress of dental science, is a lack of sufficient knowledge to progress faster—in a word, *ignorance*—the ignorance of those professing to practice dentistry primarily, and the consequent ignorance of those procuring dental operations secondarily.

Were the masses of community not ignorant of the advantages of dentistry—of the *possibilities* in dentistry, these ignorant dental pretenders, these excrescences on our young and otherwise symmetrical body, just now pulsating in every fibre with the glow of healthful progressive stimuli, would soon disappear either by excision, or resolution.

It is a trite maxim in commerce, I believe, that the supply is very speedily regulated by the demand.

I believe this law of supply and demand is the key with which to unlock the question now under consideration.

*Educate the people to demand a supply of a better quality.* Each community demands dental work consonant with previous experience, according as it has or has not received *correct* instruction.

There are two processes of instructing the community: direct and indirect. The direct consists in calling the attention of all with whom we come in contact, either personally or through the public prints, to the natural laws governing the subject we wish to teach.

The indirect consists in the making of proper exertions to restrain, and if possible, prevent the making of false statements, and hence, the formation of erroneous conclusions.

That faculty of the mind denominated *reason*, can not be exercised without an object.

A young child must know that fire exists, and something of its nature before he will refrain from placing his hand upon a hot stove.

Propositions which set the reasoning faculty of the mind at work, may be either true or false, good or evil; if false or evil, much time will necessarily be consumed, and labor expended in sifting out and isolating truth, which alone is the basis of all knowledge. For this reason if for no other, intelligent parents are always particular to never deceive their children. Much of the time of mature life is consumed in unlearning the errors of an early education.

To guard against teaching the youth of the country error and vice, laws have been generally and wisely enacted requiring teachers of public schools to provide themselves with certificates of qualification.

Indeed, this matter of fact or falsehood is considered of so great importance in this, and all other civilized countries so far as I can learn, that the making of an incorrect statement under certain circumstances, to twelve men instead of an indefinite number of children, constitutes one of the greatest criminal offenses.

Judged by this standard, where shall we place in the moral scale those who profess knowledge not possessed, and thereby obtain money under false pretenses for the *destruction* instead of preservation of valuable vital organs, in attempting to treat pathological conditions not understood?

If only one human being existed upon the face of the earth, his own fancy would be the only human law necessary for his government.

When a number of persons reside in such proximity, either close or remote, as to make it desirable to have intercourse with each other, it becomes necessary to observe, and by mutual consent establish such regulations as will prevent one person, if disposed, from interfering with the rights of another.

In this principle we observe the origin of law.

As conscientious persons entertain different views of the proper mode of arriving at the same object, and designing persons object to arriving at it by any mode, it is seldom possible to pass a law by a unanimous vote; hence, the custom of yielding the desires of the minority to the opinion of the majority.

It is no restriction upon liberty, or bar to the claims of a free government, that each individual in it can not be allowed to always follow to the extreme the dictates of his own caprice.

Such a course would speedily lead to lust, brigandage, and anarchy; and constitute a state of barbarism rather than civilization.

It is no argument against the justice of the law prescribing punishment for theft and murder, that thieves and murderers voted against it.

At Ovid, in Clinton county, Michigan, in August last, a steam boiler which had been in use only about one month exploded, and killed three men and badly mutilated a fourth, the so-called engineer.

At the time of the explosion, there was no steam gauge in use, and the lever holding down the safety valve suspended various pieces of old castings of unknown weight.

It is enough that the persons in charge of such powerful engines of death and destruction, have the ability to rake out the ashes, fill the arch with wood, and open the valve to let on steam?

If I chance to own property and occupy an office adjoining such an establishment, am I not clearly entitled to the protection accruing from a judicious management of its machinery, by a person possessing a full knowledge of all the mechanical principles and natural laws involved in the premises?

Should I not have the legal right to demand such protection?

On the other hand, should he not have the legal right to demand that I possess the requisite knowledge, before presuming to prescribe for his physical ailment?

Should we not each have the legal right to demand that our neighbor, the druggist, possess *some* knowledge—yea, a *thorough* knowledge of the remedial agents he compounds in response to the requirements of my written prescription?

I am well aware that a class of persons exists, small though it be, who maintain that such laws would tend to a species of monopoly—maintain that in our free country we should allow any person to engage in any lawful business he may desire.

The absurdity of assuming so broad a position under the cloak of freedom, was evidently appreciated by our legislative bodies in founding our common school system, when they enacted laws providing for boards of school inspectors, to ascertain and certify to the qualifications of persons desiring to teach.

The absurdity of so broad a position *should* be appreciated, by all who have observed any one of the numerous cases of death resulting from incompetent druggists and druggists' clerks dispensing valuable remedies in poisonous doses, and otherwise harmless remedies in poisonous compounds.

The school law of the State of Michigan, provides that the Superintendent of Public Instruction *may*, and the County Superintendents *shall* examine all persons offering themselves as teachers, etc.

In addition, each township has a board of school inspectors, who may not only grant certificates, but re-examine and annul them.

An appreciation of the necessity for an especial preparation for a particular business, is fully implied in the following language, which is that of school law now in force in this State:

"The Board of Instructors of the Normal School, shall give to every graduate receiving such diploma, a certificate, which shall serve as a legal certificate of qualification to teach in the primary schools of any township in this State," etc.

On page 316, of the Session Laws of the State of Michigan, for the year 1863, I find the following language:

"The people of the State of Michigan enact, That any Circuit Court may grant to any citizen of this State, of good moral character, and of the age of twenty-one years, a license to practice as an attorney and counselor at law, and solicitor and counselor in chancery, upon an examination at any term of such court, in the presence of the Circuit Judge, in open court, when satisfied that the applicant possesses sufficient legal learning and ability to discharge the duties of such office: *Provided*, That such examination shall in no case be required when said Circuit Court shall be satisfied, by the production of his diploma, or otherwise, that said applicant is a graduate of the law school of the Michigan State University."

From the language of these wise and just laws, we see that where the moral and intellectual culture of their children is concerned, in the one case, and the very mighty question of pocket, in the other, the people of the State of Michigan, in their majesty and wisdom, step forth and interpose in behalf of qualification.

As intellectual culture and pocket, are always secondary to life and generally to health, we will see what the statute provides in *this* direction.

As regards the qualification of persons in charge of stationary engines, it makes no provision whatever.

The numerous accidents resulting in a fearful destruction of life and limb, all clearly the result of incompetency, together with the great facilities our university affords for acquiring suitable knowledge in this direction, lead me to believe that this omission is clearly the result of an oversight.

With two medical colleges in our State, and one directly under its fostering care, 'tis but natural to infer that our statute law will be found such as at least to *encourage* young men to qualify themselves before engaging in the practice of the healing art.

I find the most direct, and nearly all the law relating to this question, in the following language:

"If any physician or other person, while in a state of intoxication, shall prescribe any poison, drug or medicine, to another person, he shall be punished by imprisonment in the county jail not more than one year, or by fine not exceeding five hundred dollars."

Nearly all the law we have touching the responsibility of the apothecary, is in the following language:

"Every apothecary, druggist or other person, who shall sell and deliver any arsenic, corrosive sublimate, prussic acid, or other substance or liquid usually denominated poisonous, or any tartar emetic, without having the word 'poison' and the true name thereof, written or printed upon a label attached to the vial, box or parcel, containing the same, shall be punished by a fine not exceeding one hundred dollars."

Would it not be wiser and far more beneficial,—would it not in numberless cases prevent human suffering and unnecessary sacrifice of human life, to insist upon a reasonable degree of qualification, rather than to prescribe a mode of punishment for a drunken man who in attempting to practice medicine without any knowledge of it, prescribes poison?

That engineers, druggists, physicians, surgeons and dentists, who to a great extent hold the lives of community in their hands, should be competent and considerate, I think is self-evident.

That each man, woman and child, constituting our entire community, needs protection from impending harm in each of these several directions, is obvious.

That an earnest appeal by the community to our legislature, for such protection will be successful, can not for a moment be doubted.

